# Gait characteristics in older adults using the ProtoKinetics Zeno walkway: the atherosclerosis risk in communities (ARIC) study

**DOI:** 10.3389/fnagi.2026.1901340

**Published:** 2026-09-10

**Authors:** Katherine J. Hunzinger, Laura Skow, Andrea L. C. Schneider, Michael E. Griswold, Xiaoqian Zhu, Farwa Ali, Pamela L. Lutsey, Jennifer A. Schrack, Luigi Ferrucci, Eleanor M. Simonsick, B. Gwen Windham

**Affiliations:** 1 Department of Exercise Science, Thomas Jefferson University, Philadelphia, PA, United States; 2 Department of Epidemiology, Johns Hopkins University, Baltimore, MD, United States; 3 Department of Neurology, University of Pennsylvania Perelman School of Medicine, Philadelphia, PA, United States; 4 Department of Biostatistics, Epidemiology, and Informatics, University of Pennsylvania Perelman School of Medicine, Philadelphia, PA, United States; 5 Memory Impairment and Neurodegenerative Dementia (MIND) Center, University of Mississippi Medical Center, Jackson, MS, United States; 6 Department of Neurology, Mayo Clinic, Rochester, MN, United States; 7 Division of Epidemiology and Community Health, University of Minnesota, Minneapolis, MN, United States; 8 National Institute on Aging, Intramural Research Program, Bethesda, MD, United States

**Keywords:** aging, Bland—Altman method, electronic gait mat, executive function, gait speed

## Abstract

**Background:**

Gait speed is an important indicator of functional status and health outcomes in older adults. Electronic gait mats are common in research, but stopwatches are more feasible in clinical practice. This study compared agreement between single task (ST) gait speed measured by stopwatch and gait mat and described single and dual task (DT) gait performance measured by gait mat.

**Methods:**

A subset of Atherosclerosis Risk in Communities (ARIC) Study Visit 8 participants completed ST cognitive (serial subtractions), ST gait speed, and DT gait (gait with serial subtractions) on an electronic gait mat; ST gait speed was also assessed by stopwatch. Bland Altman analyses examined agreement between ST gait speed methods. Adjusted linear mixed effects models estimated differences between ST and DT gait parameters.

**Results:**

Among 364 participants (mean 81.8 years, 54.7% female), faster gait mat gait speeds were associated with a greater discrepancy from stopwatch measurements (*β*: −0.24 [95%CI: −0.31, −0.17]), indicating stopwatch underestimation. Participants exhibited mutual interference under DT, walking slower (mean difference in gait speed: −25.0 cm/s [95%CI: −26.8, −23.2]) with shorter (mean difference in step length: −6.2 cm [95%CI: −6.8, −5.6]) and wider steps (mean difference in stride width: 1.6 cm [95%CI: 1.3, 1.9]).

**Conclusion:**

Gait mat and stopwatch measurements showed acceptable agreement, supporting clinical use. However, greater disagreement at faster speeds suggests gait mats may be more appropriate for faster walking speeds. These findings support future investigations of ST and DT gait performance within ARIC and other aging cohorts, including relationships with cognitive decline, mobility limitations, and adverse health outcomes.

## 1 Introduction

Single task gait, mainly walking speed, is considered an important marker of overall health status in older adults and has been associated with balance, functional ability (Fritz and Lusardi, 2009), cognitive status (Yogev et al., 2009), postural control (Woollacott and Shumway-Cook, 2002), mental health status (Lemke et al., 2000; Middleton et al., 2016), hospitalization (Montero-Odasso et al., 2005), and death (Hardy et al., 2007), among other outcomes. As such, monitoring gait as individuals age is an important, although underutilized, metric for clinicians (Hardy et al., 2007; Verlinden et al., 2013; Montero-odasso et al., 2017). Typically, gait speed is measured subjectively in clinical practices or over a short course (e.g., 4–6 meters) with a stopwatch in most research studies (Woollacott and Shumway-Cook, 2002; Hardy et al., 2007). Stopwatch-based gait assessments are inexpensive, clinically feasible, and widely implemented, making them clinically feasible for routine practice and large epidemiologic studies. However, stopwatch assessments only provide a measure of gait speed and do not capture additional spatiotemporal gait characteristics (e.g., gait initiation/termination phases, step width/length) that may provide further insights into mobility impairments, fall risk, and cognitive decline. Electronic gait mats can quantify these biomechanical gait characteristics while also measuring gait speed (Lord et al., 2013). Previous studies have generally reported acceptable agreement between stopwatch- and gait mat-derived gait speed; however, these studies have primarily included small samples or clinical populations (e.g., persons with prior stroke, neurological disease, or musculoskeletal disease), limiting their generalizability to community-dwelling older adults. Moreover, few investigations have evaluated agreement while simultaneously characterizing the broader range of spatiotemporal gait parameters and dual-task gait performance that electronic gait mats uniquely provide (Stuck et al., 2020).

Purposeful community locomotion requires concurrent cognitive tasks such as attention; selective attention specifically allows one to focus on important stimuli and ignore irrelevant distractors. In addition, people often engage cognitively while walking, such as thinking about future actions, conversing, or holding an object (Al-yahya et al., 2011). Thus, gait with a concurrent task (i.e., dual task gait) places a larger demand on cognitive and sensory systems, requiring more executive functioning skills than single task gait (Sheridan and Hausdorff, 2007). Dual task gait employs frontal lobe [i.e., dorsolateral prefrontal cortex (Al-yahya et al., 2011)] executive functioning to enable processing of concurrent cognitive and motor demands (Yogev et al., 2009). The demands placed on the cognitive and motor systems during locomotion can be investigated using dual task methods. Specifically, dual task function is commonly measured as dual task cost, that is, the difference in performance between undivided (i.e., single-task) and divided (i.e., dual task) attention conditions, typically expressed as a percentage of the single task performance (Howell et al., 2018). Measures of dual task cost have been shown to differentiate normal subjects from those with a mild traumatic brain injury (Howell et al., 2013) or mild cognitive impairment (MCI) (Li and Harmer, 2020), and are predictive of future injury (Howell et al., 2018) and incident MCI (Ramírez and Gutiérrez, 2021) in older adults with normal single task gait. Dual task assessments provide a method to identify where performance is impacted during dual task conditions (i.e., cognitive task or motor task), gain insights into task prioritization (Al-yahya et al., 2011) and potentially improve individualized risk assessments for tailored gait rehabilitation (Varela-Vásquez et al., 2020).

Comprehensive single task and dual task gait assessments remain relatively uncommon in large longitudinal epidemiologic cohorts of community-dwelling older adults, limiting opportunities to characterize these measures and evaluate their utility for aging research. Given the utility of gait as a measure of overall health, incorporating detailed gait assessments into longitudinal cohorts provides a valuable foundation for investigating future associations with cognitive decline, mobility limitations, and other aging-related outcomes. Gait mats provide a convenient and largely portable method for biomechanical analysis of gait, but they have not been extensively used in established longitudinal cohort studies, and agreement with traditional stopwatch-derived gait speed has not been well characterized in large community-based populations. Thus, this study aimed to compare the level of agreement between gait speed measured by stopwatch and gait mat among community dwelling older adults while also describing single and dual task performance measured by gait mat among community dwelling older adults in the Atherosclerosis Risk in Communities (ARIC) Study.

## 2 Methods

### 2.1 Participants

The ARIC Study is a deeply phenotyped, ongoing longitudinal study of initially middle-aged adults from four communities in the United States (Forsyth County, NC; Jackson, MS; Washington County, MD; northwest suburbs of Minneapolis, MN). Detailed study design and protocol have been previously described (ARIC Investigators, 1989; Wright et al., 2021). In late life (Visit 5, 2011–2013), ARIC began collecting gait data using a stopwatch (Wright et al., 2021); a gait mat was added to the in-person assessment protocol at Visit 8 (2020). The present analyses used only Visit 8 assessments and did not apply additional inclusion or exclusion criteria based on medical comorbidities; thus, stopwatch-derived and gait mat-derived gait speed were compared using data collected at the same timepoint (i.e., ARIC Visit 8). Participants were eligible to be included in the present analysis if they were alive at the start of the in-person ARIC Visit 8 (*n* = 7,083), which began in January 2020 but transitioned to a telephone visit in March 2020 due to the COVID pandemic. A total of 451 participants attended Visit 8 in-person prior to the COVID pandemic-related shutdown, of whom 390 participants underwent gait assessment. Of these 390 participants, we excluded 26 participants who required the use of an aid during the gait trials, resulting in a total of 364 participants in the current analyses (included participants had single task stopwatch and/or single task gait mat and/or dual task gait mat data). The number of participants contributing data differed by assessment method and testing condition. Gait data were available for 364 participants for the single-task gait mat assessment, 329 participants for the dual-task gait mat assessment, and 235 participants for the single-task stopwatch assessment. All participants or legally authorized proxies provided written informed consent, and the study was approved by the institutional review board of each participating institution. The ARIC Study was approved by the respective institutional review boards at each of the collaborating institutions.

### 2.2 Single task stopwatch-derived gait speed

All gait assessments were conducted with participants wearing comfortable shoes (e.g., no high heels) from a standing position as previously described (Windham et al., 2017). Consistent with the standardized ARIC gait assessment protocol, participants were offered two gait trials to improve the reliability of gait speed assessment, with the faster of the two trials used for analyses (Windham et al., 2017). If a participant was unable to safely complete a second trial (e.g., due to fatigue, mobility limitations, balance concerns, or other testing constraints), the available single trial was used. Participants were asked to walk at their usual pace along a 4-meter course marked in an unobstructed corridor and to continue walking past the finish line into a deceleration zone (≥1 m) before slowing. The deceleration zone was incorporated to encourage participants to maintain their usual walking pace through the time assessment rather than slowing in anticipation of the finish line, consistent with recommendations for gait speed measurement (Johnson et al., 2020; Jarvis et al., 2021). Participants walked on a verbal cue to begin. Stopwatch timing began with the first movement by the participant and ended when the participant’s foot crossed a vertical plane at the finish line. Times were recorded to the nearest hundredth of a second.

### 2.3 Gait mat: single and dual task gait

Gait was captured using a ProtoKinetics Zeno™ 21-foot Walkway floor pressure sensor system with a 5-meter active sensor zone. Participants completed the same standardized 4-meter walking task used for the stopwatch assessment. The additional length of the gait mat allowed participants to continue walking beyond the measured distance before slowing, consistent with the deceleration zone incorporated into the stopwatch protocol. Each usual pace gait trial used the same protocol as the single task stopwatch gait speed measure. The standardized protocol facilitated comparison between gait speed measured by the stopwatch and the gait mat. Visual review and quality control of all gait mat data was performed by an experiences rater, who examined individual footfalls to remove extraneous steps (e.g., from canes or multiples passes) and relabel any mislabeled right/left steps. Instructions were provided with participants’ feet on the mat, which could vary in time across participants; data were extracted beginning with the first footfall.

For single task cognition, participants serially subtracted 3 beginning at 100 while stationary. If participants could not successfully subtract by 3 s in the single task condition, the examiner offered subtractions by 2 s. This provided a gradient of ability for participants whether they could complete serial 3 or serial 2 subtraction rather than assigning all who were unable to perform serial 3’s a score of zero (McPhee et al., 2022). If an error was made but the subsequent subtraction was correct, the correct calculation following an error was counted as correct. Single task cognitive outcome was reported as the total number of correct subtractions to a maximum of seven. Among participants completing the gait mat assessments, cognitive data (serial 3 s or serial 2 s) were missing for 41 (11.3%) single-task and 39 (11.9%) dual-task assessments. For single task gait, participants were instructed to walk at their usual pace on the mat using the same instructions as the stopwatch-derived gait speed protocol.

For dual task conditions, participants were asked to follow the same instructions as the single task gait test while concurrently calculating the same serial subtractions that were done during ST, using the same instructions provided during single task. Dual task cost was calculated for each gait outcome using the following equation to express change from the single task condition as a percentage: |(Dual Task - Single Task)/(Single Task)|*100% (where higher positive values indicate greater dual-task cost) (Plummer and Eskes, 2015).

Gait parameters, representing spatial, temporal, temporophasic, and spatiotemporal outcomes commonly used in the gait literature were selected *a priori* (Oh-Park et al., 2010; Hollman et al., 2011; Smith et al., 2016; Ramírez and Gutiérrez, 2021). These outcomes were calculated by ProtoKinetics™ PKmas software (Version 5). Definitions utilized are provided in Table 1.

**Table 1:** Operational definitions of single and dual task gait parameters, ARIC visit 8 (2020).

Gait parameters	Operational definition
Spatial	
Step length (cm)	Distance between corresponding successive points on the heel of opposite feet measured parallel to the direction of progression for the ipsilateral stride of which it is the second part
Stride width (cm)	Distance between a line connecting the two ipsilateral foot heel contacts (the stride) and the contralateral foot heel contact between those events and is measured perpendicular to the stride
Stride length (cm)	Distance from the heel of one foot to the following heel of the same foot
Temporal	
Cadence (steps/min)	The number of footfalls minus one, divided by the ambulation time
Single support time (sec)	The period of time when only the current foot is in contact with the ground
Double support time (sec)	The sum of all periods of time when both feet are in contact with the ground during stance phase
Gait cycle time (sec), or Stride Time (sec)	The period of time from first contact of one foot to the following first contact of the same foot
Stance time (sec)	The period of time when the foot is in contact with the ground
Step time (sec)	The period of time taken for one step and is measured from first contact of one foot to the first contact of following other foot
Swing time (sec)	The period of time when the foot is not in contact with the ground
Temporophasic	
% Stance	Stance time presented as a percentage of the gait cycle time
% Swing	Swing time presented as a percentage of the gait cycle time
% Double support	Double support time presented as a percentage of gait cycle time
Spatiotemporal	
Gait speed (m/s)	The velocity is obtained after dividing the sum of all stride length, by the sum of all stride time

### 2.4 Other variables

Participants self-reported age, sex (male; female), race (American/Alaskan Indian; Asian; Black; White), and education (<high school; high school, GED, or vocational school; any college or higher) at Visit 1 (1987–1989). Consistent with ARIC cohort design, analyses were limited to participants who self-identified as Black or White, as individuals from other racial groups and Black participants recruited at the Minnesota and Maryland field centers were excluded from ARIC analytic datasets due to race/center aliasing; thus all participants included in the Visit 8 subset self-reported race as Black or White (ARIC Investigators, 1989; Wright et al., 2021). Body mass index (BMI) was calculated as weight (kg) divided by height squared (m^2^) using the most recent height and weight measurements at ARIC Visit 7, the most recent visit in which height and weight were collected prior to Visit 8. Height and weight were not collected at Visit 8 due to study modifications during the COVID-19 pandemic.

### 2.5 Statistical analysis

Participant characteristics are shown for the three different measurement methods (single task gait mat, single task stopwatch, and dual task gait mat) as means and standard deviations (SDs) for continuous variables and as numbers and percentages for categorical variables.

To estimate and compare average gait parameters across different measurement methods, we utilized linear mixed models with random intercepts and Huber-White robust standard errors. Gait measures from the three measurement methods were recorded as a single outcome variable with multiple rows per participant and a separate variable indicating measurement method served as the primary exposure variable. Individual gait parameters were analyzed in separate models. All models were adjusted for age, sex, BMI, race/center, and education. Post-estimation marginal standardization was used to obtain the adjusted average gait speed for each measurement method. We then calculated the absolute difference and dual task cost in marginal means to quantify the average difference in gait speed between methods (Localio et al., 2025). Linear mixed-effects models used all available outcome measurements under the assumption that data were missing at random. Participants were not required to have complete data across all assessment methods or testing conditions to be included in the analyses.

For participants with stopwatch single task and gait mat single task measures, agreement between methods was evaluated using Bland–Altman analyses. Mean bias, 95% limits of agreement, and the intraclass correlation coefficient (ICC) were calculated. Modified Bland–Altman plots were used to compare gait speed. Specifically, we plotted the differences between the two measurements (stopwatch minus gait mat) against gait mat and separately against stopwatch measurements, adjusting for age, sex, BMI, race/center, and education. To aid visualization of potential trends, we overlaid both a locally weighted scatterplot smoothing (LOWESS) curve and a linear regression line on each plot.

To examine task prioritization for dual task gait, we created a scatterplot of each individual’s gait speed dual task cost versus cognition dual task cost, where dual task cost was defined as (Dual Task – Single Task)/(Single Task)*100%. To illustrate the joint distribution of gait and cognitive dual task costs, we added contour lines representing the 10th, 25th, 50th, and 75th percentiles.

Analyses were performed using Stata SE (Version 18, StataCorp, College Station, TX) with a two-sided α level of 0.05 being considered statistically significant.

## 3 Results

### 3.1 Participant characteristics

Among 364 participants included in the analyses (mean age of 81.8 years, 54.7% female, 19.0% Black race), all 364 had gait mat single task gait data, 235 had stopwatch single task gait data, and 329 had dual task gait mat data (Table 2). Overall, the average adjusted single task gait mat gait speed was 102.97 cm/s (95% CI: 101.15, 104.78). Compared to single task gait mat, gait speed measured via stopwatch for single task conditions and gait speed under dual task conditions were slower (−7.5 cm/s, 95% CI: −9.1, −6.0 and −25.0 cm/s, 95% CI: −26.8, −23.2, respectively) (Supplementary Table 1).

**Table 2:** Participant characteristics, ARIC visit 8 (2020), *N* = 364.

Participant characteristic	Single Task - Gait Mat Population (*n* = 364)	Single Task – Stopwatch Population (*n* = 235)	Dual Task - Gait Mat Population (*n* = 329)
Age (years), mean (SD)	81.8 (4.3)	81.6 (4.3)	81.8 (4.3)
Male sex, *n* (%)	165 (45.3)	95 (40.4)	159 (48.3)
Body mass index (kg/m^2^)*, mean (SD)	28.4 (4.8)	28.4 (4.6)	28.3 (4.9)
Education, *n* (%)			
> 12 years	163 (44.8)	93 (39.6)	160 (48.6)
12 years, vocational, or equivalent	167 (45.9)	112 (47.7)	147 (44.7)
< 12 years	34 (9.3)	30 (12.8)	22 (6.7)
Race/center, *n* (%)			
Washington County, Maryland, White	104 (28.6)	102 (43.4)	93 (28.3)
Minneapolis, Minnesota, White	115 (31.6)	10 (4.3)	113 (34.3)
Jackson, Mississippi, Black	69 (19.0)	69 (29.4)	53 (16.1)
Forsyth County, North Carolina, Black	1 (0.3)	1 (0.4)	1 (0.3)
Forsyth County, North Carolina, White	73 (20.1)	51 (21.7)	69 (21.0)
Step length (cm), mean (SD)	57.9 (9.6)	--	52.4 (10.8)
Stride width (cm), mean (SD)	8.7 (3.9)	--	10.3 (4.2)
Stride length (cm), mean (SD)	115.7 (19.2)	--	104.7 (21.6)
Cadence (steps/min), mean (SD)	106.1 (9.8)	--	89.6 (17.1)
Single support (sec), mean (SD)	0.4 (0.0)	--	0.4 (0.1)
Double support (sec), mean (SD)	0.4 (0.1)	--	0.5 (0.3)
Stride time (sec), mean (SD)	1.1 (0.1)	--	1.4 (0.4)
Stance time (sec), mean (SD)	0.7 (0.1)	--	1.0 (0.3)
Step time (sec), mean (SD)	0.6 (0.1)	--	0.7 (0.2)
Swing time (sec), mean (SD)	0.4 (0.0)	--	0.4 (0.1)
Stance %, mean (SD)	65.9 (2.3)	--	67.6 (3.5)
Swing %, mean (SD)	34.1 (2.3)	--	32.4 (3.5)
Double support %, mean (SD)	31.1 (4.7)	--	34.9 (7.3)
Gait speed (cm/s), mean (SD)	103.2 (20.0)	92.9 (18.2)	79.5 (24.1)
Cognition: serial 3 s or serial 2 s* (% correct), mean (SD)	81.3 (16.7)	--	74.6 (24.6)

*BMI collected at ARIC Visit 7.

### 3.2 Comparison of stopwatch and gait mat single task gait speed

**Figure 1:**
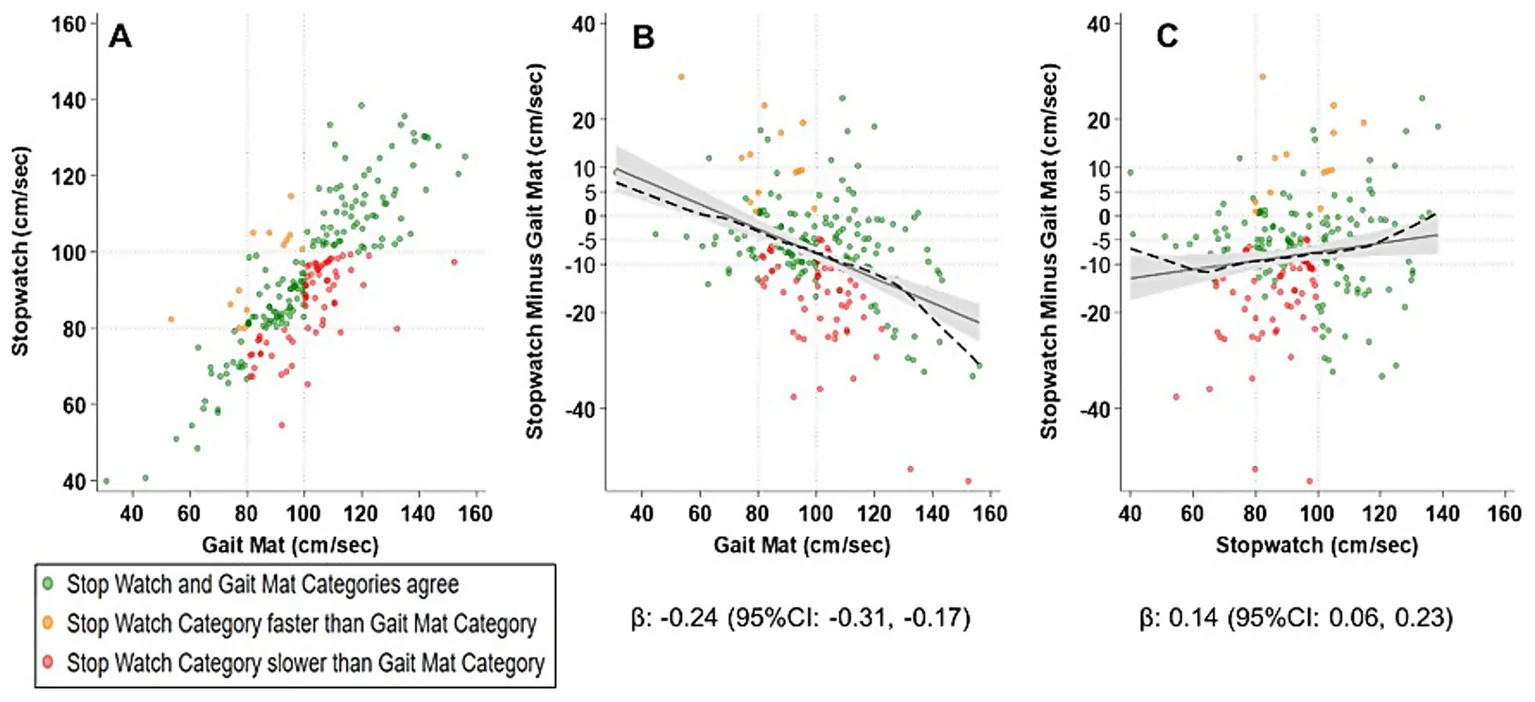
Bland Altman plots comparing Stopwatch to ProtoKinetics Motional Analysis Software: Zeno Gait Mat for single task usual gait speed, ARIC Visit 8 (2020), *n* = 235. **(A)** Concordance between stopwatch and gait mat measured gait speed and **(B,C)** adjusted Bland Altman plots comparing stopwatch and gait mat measured gait speed (adjusted for age, sex, BMI, race/center, and education). Individual data are color coded by clinically relevant timepoints of 80 cm/s and 100 cm/s creating graphical quadrants, whereby if an individual's gait mat measured gait speed and stopwatch measured gait speed placed them in the same quadrant, they were color coded green. Conversely, if the stopwatch placed the participant in a faster quadrant, it was coded yellow and if the stopwatch placed the participant in a slower quadrant, it was coded red.

Figure 1A illustrates agreement of single task gait speed by gait mat and stopwatch. The color scheme represents classification of single task gait speed by gait mat and stopwatch using clinically relevant cutoffs of 80 cm/s and 100 cm/s (green: same clinical category by gait mat and stopwatch, red: stopwatch slower than gait mat, orange: stopwatch faster than gait mat) (Cesari et al., 2005; Studenski, 2011). Adjusted associations revealed that 28.1% of participants were miscategorized into a slower gait speed category when using stopwatch measurements compared to the gait mat measurement. Overall, 66.4% of participants were classified into the same clinically relevant gait speed category by both assessment methods, with the greatest discordance observed among participants with gait speeds >100 cm/s. Figures 1B,C show adjusted Bland–Altman plots comparing single task gait speed velocity from the gait mat and stopwatch. Bland–Altman analysis demonstrated a mean difference (gait mat – stopwatch) of 8.1 cm/s, with 95% limits of agreement ranging from −14.6 to 30.8 cm/s. Gait mat derived gait speed was associated with the difference between stopwatch and gait mat measurements (*β*: −0.24, 95%CI: −0.31, −0.17; *p* < 0.001), whereby faster gait mat-derived gait speeds corresponded to slower stopwatch-derived gait speeds (i.e., underestimation). Conversely, gait speed measured by the stopwatch was positively associated with the difference between the two methods (*β*: 0.14, 95%CI: 0.06, 0.23; *p* = 0.001), indicating that higher stopwatch-measured gait speeds corresponded to a greater overestimation of speed relative to the gait mat.

### 3.3 Characterization of gait mat measured single task and dual task gait parameters

**Figure 2:**
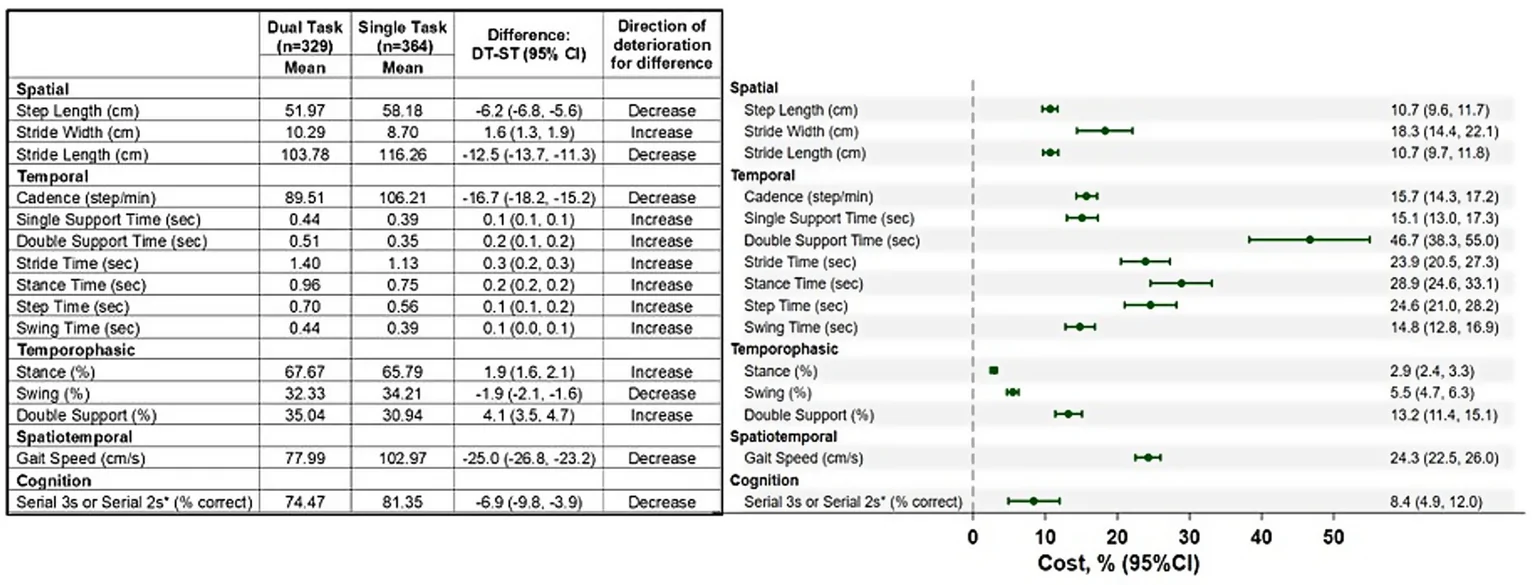
Single and dual task gait and cognitive parameters and dual task cost, ARIC Visit 8 (2020). Estimates were obtained from linear mixed effects models adjusted for age, sex, body mass index, race/center, and education (each row represents a separate model).

The average adjusted single task gait, dual task gait, cognitive parameters, and dual task cost are presented in Figure 2; The difference between single task and dual task as well as the direction of deterioration (i.e., whether a positive or negative difference is indicative of worse performance) are also presented. Overall, participants exhibited a more conservative gait strategy under dual task compared to single task conditions, walking slower (mean difference in gait speed: −25.0 cm/s [95%CI: −26.8, −23.2]) with shorter (mean difference in step length: −6.2 cm [95%CI: −6.8, −5.6]) and wider (mean difference in stride width: 1.6 cm [95%CI: 1.3, 1.9]) steps, while spending a greater proportion of gait in double support (mean difference: 4.1% [95%CI: 3.5, 4.7]). The greatest dual task cost were observed in double support time (46.7, 95%CI: 38.3, 55.0), followed by stance time (28.9, 95%CI: 24.6, 33.1), step time (24.6, 95%CI: 21.0, 28.2), gait speed (24.3, 95%CI: 22.5, 26.0), and stride time (23.9, 95%CI: 20.5, 27.3).

**Figure 3:**
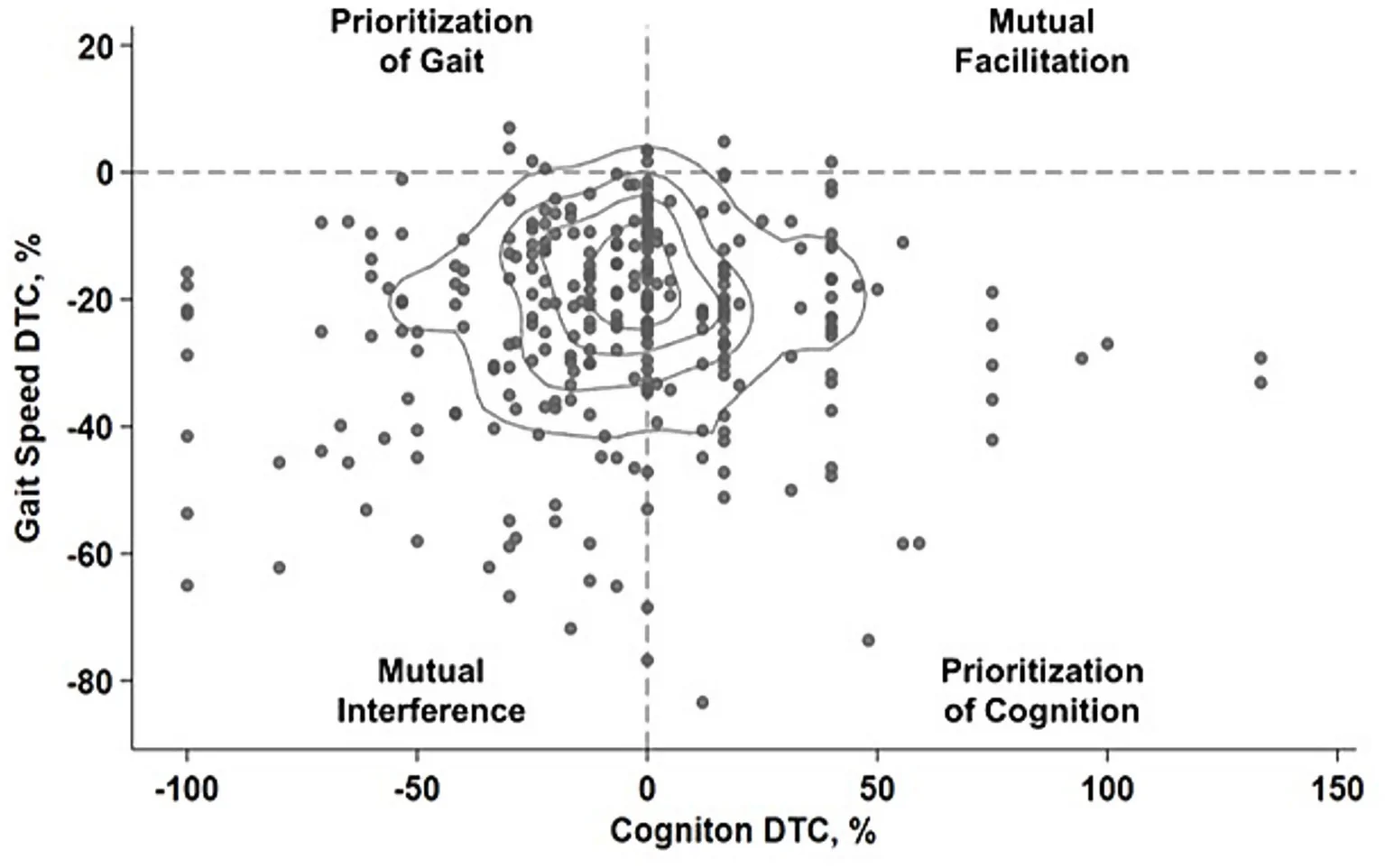
Task prioritization for dual task gait. Contour lines represent 10th, 25th, 50th, and 75th percentiles. For graphing purposes, dual task cost (DTC) for gait speed and cognition were calculated as (Dual Task - Single Task)/(Single Task)*100% and plotted to identify task prioritization (e.g., mutual interference whereby both motor and cognitive task performance decline under dual task conditions, mutual facilitation whereby both improve under dual task conditions, or prioritization of either cognition or gait).

Task prioritization in dual task gait is presented in Figure 3. Overall, this cohort predominantly displayed cognitive interference on gait, whereby gait performance worsened under dual task conditions (80.9%). Moreover, 50.3% of participants displayed mutual interference, whereby gait speed slowed with more subtraction errors under the dual task condition. Two individuals appeared to display mutual facilitation; however, this was explained by these participants not answering any subtractions correctly under the single task cognitive condition but answering at least one correctly under the dual task condition.

## 4 Discussion

This study had two primary aims: (1) to compare agreement between stopwatch- and gait mat-derived gait speed, and (2) to describe single task and dual task gait performance in a relatively large cohort of Black and White community-dwelling adults 75 years and older in the ARIC study. Overall, single task gait speed measured by the PKMas software and stopwatch methods demonstrated acceptable agreement in this population (mean difference: 8.1 cm/s; 95% limits of agreement: −14.6 to 30.8 cm/s). However, adjusted Bland–Altman plots revealed some systematic differences in measurement across the range of gait speeds, whereby faster gait speeds recorded by the gait mat tended to be underestimated by the stopwatch, while slower stopwatch-recorded speeds tended to overestimate gait speed relative to the gait mat, potentially due to measurement error related to reaction times of the examiner using the stopwatch. The majority of participants also demonstrated mutual interference during a dual task condition, whereby they performed worse on both the motor (i.e., gait) and cognitive (i.e., serial subtraction) tasks. These findings provide important implications for interpretation of gait speed thresholds and establish reference data for future investigations of single and dual task gait performance within the ARIC study.

One important purpose of this study was to compare the level of agreement between gait speed assessment methods (gait mat and stopwatch). When categorizing participants into clinically relevant gait speed bins (i.e., ≤80 cm/s, >80 to <100 cm/s, and ≥100 cm/s), only 66.4% were categorized in the same bins between methods, with the largest misclassification (28.1%) occurring at faster gait mat measured gait speeds (i.e., ≥100 cm/s). Additionally, higher (i.e., faster) stopwatch measured gait speeds corresponded to a greater overestimation of speed relative to the gait mat. The slower stopwatch measured gait speeds may be due in part to measurement latency associated with an examiner’s visual observation of the participant initiating movement or crossing the end of the gait analysis zone, whereas the gait mat uses footfall data to determine walking speed in the measurement zone. This is consistent with prior research reporting faster gait mat measured gait speeds than stop watch measured gait speeds (Youdas et al., 2006). As a result of this latency, the time measured by stopwatch would result in a longer time to walk 4 meters, resulting in a slower calculated gait speed (i.e., 4 meters divided by time). This is consistent with the patterns observed in the Bland–Altman analyses whereby the stopwatch gait speed at faster gait mat derived speeds was slower (i.e., smaller number) resulting in the negative mean difference. Importantly, gait speeds less than 0.6 m/s, a suggested threshold for dysmobility that is also a sensitive marker of physical frailty (Cummings et al., 2014), were similar when using a stopwatch or gait mat. Therefore, for frail older adults, a stopwatch-derived gait speed may be adequate and it is more feasible in clinical settings. Conversely, gait mat derived gait data may be more appropriate for community-dwelling older adults with faster walking speeds. Importantly, in higher-functioning older adults, gait mat-derived assessments may be more sensitive to change, which may translate to improved opportunities for earlier intervention efforts. Beyond informing gait assessment in individuals at the extremes of walking performance, these findings also have implications for population-based screening. Given that gait speed is increasingly recognized as a biomarker of overall health and future adverse outcomes (e.g., falls, hospitalization), even modest differences in gait speed measurement between assessment methods may influence risk stratification or clinical decision-making when established gait speed thresholds are used to identify older adults at increased health risk (Fritz and Lusardi, 2009; Middleton et al., 2016). Longitudinal data are needed for verification and further assessment.

This study extends existing work by reporting spatiotemporal parameters in a large, representative sample of community-dwelling older Black and White adults over the age of 75 years who were not selected according to body habitus or prevalence of health conditions such as hypertension and diabetes. Consistent with other studies of older adults (Hausdorff et al., 2008; Smith et al., 2016; Lara et al., 2024), this cohort walked more conservatively under dual task conditions evidenced by performance declines (i.e., dual task cost) in gait speed (24.3%), reductions in stride length (10.7%), and increases in stride width (18.3%) and percent time in double support (46.7%). These reductions are consistent with previous studies in older adults without neurological impairments and can be characterized as mutual interference whereby the motor task (i.e., gait) and the cognitive task (i.e., serial subtraction correct responses) performance both worsened under dual task conditions (Hausdorff et al., 2008; Plummer and Eskes, 2015; Zukowski et al., 2021). Conversely, a smaller proportion of participants exhibited cognitive only or motor only prioritization (31.9%), suggesting that most older adults in this cohort were unable to preserve performance in either domain when faced with competing demands (Plummer and Eskes, 2015; Fino et al., 2018). This conservative gait strategy is likely in response to the increased attentional demands imposed by the addition of the cognitive task relative to the single task (gait only) condition (Catena et al., 2009). Characterization and identification of gait phenotypes among community dwelling older adults has been suggested as a surrogate marker in improving the diagnosis of dementia (Allali et al., 2016). Future studies should investigate task prioritization over time and as it relates to specific aging-related conditions.

This study has important strengths and limitations. One limitation is the small sample size with gait data relative to the overall ARIC study sample as a result of the switch from in-person to telephone-based visits in the setting of the COVID pandemic. Nevertheless, this study is among the largest with gait mat and stopwatch-derived gait data, particularly in a community-dwelling older cohort. Second, although stopwatch and gait mat assessments were collecting during the same Visit 8 in-person study visit, the stopwatch gait trials were separate from the gait mat trials. Therefore, while fatigue is unlikely to have affected our data, there may still exist within-participant variability between stopwatch timed gait speed and gait mat derived gait speed that we were not able to account for in the analyses. Additionally, our findings are specific to the standardized ARIC gait assessment protocol and may not be directly generalizable to studies using different protocols or walkway configurations. Moreover, generalizability may be limited across more ethnically and racially diverse populations, as included participants are community-dwelling older Black and White adults from four US communities. We believe this study is unique in the availability of a stopwatch gait assessment protocol that was administered following centralized training at protocol inception across four US communities, followed by ongoing quality control and re-training onsite by a certified examiner at follow-up visits, including visit 8. Finally, the gait mat assessment was conducted following in-person training (live or virtual) by a single trainer along with a software and gait analysis expert who trained in-person and monitored gait data, performed visual review, cleaning, and processing of all gait data with re-training as needed for quality assurance across the four sites.

## 5 Conclusion

In summary, our findings suggest that an electronic gait mat may be more appropriate for use with some older adults, especially those with faster walking speeds. Moreover, these data pave the way for future studies within ARIC and other cohorts of older individuals to investigate the relationships between various conditions and gait performance. Larger cohorts are needed to compare electronic gait mat-derived gait assessments to stopwatch-derived gait speed in the context of clinical and research outcomes.

## Data Availability

The data analyzed in this study were obtained from the Atherosclerosis Risk in Communities (ARIC) Study and are not owned by the authors. The authors are therefore not authorized to make the data publicly available. Data may be requested through the established ARIC data access procedures, subject to applicable data use agreements, participant consent, and approval requirements. Inquiries can be directed to the corresponding author/s.
