# Supplementary material for: Gait characteristics in older adults using the ProtoKinetics Zeno walkway: the atherosclerosis risk in communities (ARIC) study
Source: Front Aging Neurosci. 2026 Sep 10;18:1901340. doi: 10.3389/fnagi.2026.1901340 (PMC13601270; doi:10.3389/fnagi.2026.1901340)
Supplement: Supplementary file 1 [file Table_1.DOCX]

**sTable1.** Comparison of Gait Speed on Each Task.

| **Task** | **Average Gait Speed (95%CI),  cm/sec** | **Difference in Gait Speed (95%CI),  cm/sec** |
| --- | --- | --- |
| Single Task: Gait Mat | 102.97 (101.15, 104.78) | Reference |
| Single Task: Stopwatch | 95.45 (93.45, 97.46) | -7.5 (-9.1, -6.0) |
| Dual Task: Gait Mat | 77.99 (75.67, 80.32) | -25.0 (-26.8, -23.2) |

Estimates were obtained from a linear mixed effects model with a random intercept; each model was adjusted for age, sex, body mass index, race/center, and education.
